# Disclosing conflicts of interest in German publications concerning health services research

**DOI:** 10.1186/1472-6963-7-78

**Published:** 2007-06-01

**Authors:** Nils Schneider, Heidrun Lingner, Friedrich W Schwartz

**Affiliations:** 1 Department of Epidemiology, Social Medicine and Health System Research, Hannover Medical School, Carl-Neuberg-Str. 1, D-30625 Hannover, Germany

## Abstract

**Background:**

The influence of the pharmaceutical industry and other stakeholders on medical science has been increasingly criticised. When dealing with conflicts of interest in scientific publications it is important to ensure the best possible transparency. The objective of this work is to examine the disclosure practice of financial and non-financial conflicts of interest in German language publications concerning health services research for the first time.

**Methods:**

We performed a systematic literature search in the PubMed data base using the MeSH term "health services research". The review was conducted on July 10, 2006, setting the limits "dates: published in the last 2 years" and "languages: German" (only articles with abstracts). 124 articles in 31 magazines were found. In the magazines the instructions for authors were examined as to whether a statement on conflicts of interest is expected – and if, in which form. Regarding the articles in the journals which require a statement, we examined whether the statement is explicitly published. The results are descriptively represented.

**Results:**

13 magazines (42%) do not require any statement on conflicts of interest, whereas 18 journals (58%) expect a statement. Two of these 18 magazines refer explicitly to the uniform requirements of the *International Committee of the Medical Journal Editors *(ICMJE); the remaining 16 magazines give differently accentuated instructions on how to disclose conflicts of interest, whereby the focus is primarily on financial issues. A statement on conflicts of interest is explicitly published in 11 of the 71 articles (15%) which are found in the magazines that require a statement with the submission of a manuscript. Related to the total number of included articles, this means that the reader explicitly receives information on potential conflicts of interest in 9% of the cases (11 of 124 articles). Statements of others that are involved in the publication process (reviewers, editors) are not available in any of the articles examined.

**Conclusion:**

A better sensitization for possible conflicts of interest in German publications concerning health services research is necessary. We suggest tightening the criteria for disclosure in the instructions for authors in the scientific journals. Among other things the equivalent consideration of financial and non-financial conflicts of interest as well as the obligatory publication of the statements should be part of good practice.

## Background

The influence of industry on medical sciences has been increasingly criticised [1,2]. One reason is that studies sponsored by the industry much more frequently produce positive results for products of the sponsor, compared to studies that are not sponsored by the industry [3,4]. For example, data exist which show that statements on cost effectiveness of diagnostic or therapeutic procedures are more frequently favourable if the investigations are sponsored by the industry [5]. The transfer of scientific expertise to clinical practice may be affected by the relationship between the authors of clinical practice guidelines and the pharmaceutical industry: four out of five authors have connections to the pharmaceutical industry, and two of these four are close advisors or employees of the companies which drugs they recommend in the guidelines [6].

This – more or less pronounced – proximity of scientists to the industry is reflected in the scientific magazines as is exemplarily highlighted by the statement of a former editor of the *New England Journal of Medicine*: one rarely finds psychiatrists for editorials on the treatment of depression who have no financial connections to the pharmaceutical industry [2,7].

A conference on "disease mongering" in 2006 [8] illustrates the importance of the topic for health services research. Among other things, it becomes clear that the interests of third parties can aim at the opening or extension of a market for a product or a service. For example the strategies can consist of the redefinition of limits and treatment objectives as well as of the influence on the public view in respect to the appropriateness of medical services; strategies that were already described by the American author Payer almost 25 years ago [9].

### Standards for disclosing conflicts of interest

An important instrument in dealing with conflicts of interest scientifically is ensuring best possible transparency [5]. Internationally accepted standards for this are set by the *Uniform Requirements for Manuscripts Submitted to Biomedical Journals *by the International Committee of Medical Journal Editors (ICMJE). Apart from financial interest, problems that may result from non-financial conflicts of interest are explicitly mentioned [10]:

" [...] Conflict of interest exists when an author (or the author's institution), reviewer, or editor has financial or personal relationships that inappropriately influence (bias) his or her actions (such relationships are also known as dual commitments, competing interests, or competing loyalties). These relationships vary from those with negligible potential to those with great potential to influence judgment, and not all relationships represent true conflict of interest. The potential for conflict of interest can exist whether or not an individual believes that the relationship affects his or her scientific judgment. Financial relationships, (such as employment, consultancies, stock ownership, honoraria, paid expert testimony) are the most easily identifiable conflicts of interest and the most likely to undermine the credibility of the journal, the authors, and of science itself. However, conflicts can occur for other reasons, such as personal relationships, academic competition, and intellectual passion. All participants in the peer review and publication process must disclose all relationship that could be viewed as presenting a potential conflict of interest. [...]".

Lack of or incomplete disclosure of potential conflicts of interest prevent the reader from applying the appropriate scepticism in the scientific dialogue [11].

### Research question

There have been no studies so far on the disclosure practice of conflicts of interest in scientific journals within the field of health services research. Therefore, the objective of this study is to examine the current practice using the example of German language journals. In particular, the following research questions should be investigated:

▪ What criteria are set up for the disclosure of conflicts of interests in the instructions for authors?

▪ How transparent is the practice of disclosure for the readers?

### Health services research in Germany

Internationally, health services research in Germany^1 ^is in an early stage of development. However, it has increasingly received attention and support especially within the last few years. Milestones are, for example, the research programs by the Federal Ministry for Education and Research (BMBF) and the compulsory health insurance funds in the year 2000 and the strategies to promote research by the German Medical Association (Bundesaerztekammer) in 2005. Quite recently Pfaff und Kaiser, who are well-known experts in this area, looked critically at the situation of health services research in Germany [12]. They state that research activities are characterized by a concentration on topics guided by special interests of stakeholders. The authors attribute this to the high proportion of commissioned research aiming at fast, practical usability and threatening the scientific soundness of health services research.

## Methods

To identify both articles and journals, a systematic literature search was conducted in the PubMed data base using the MeSH term "health services research" as well as the free terms "health system research" and "health research" which are also in use within the field of interest in Germany. The review was conducted on July 10, 2006, setting the defined PubMed limits "dates: published in the last 2 years" and "languages: German". Only articles with abstracts were included.

124 articles in 31 different journals were found (table 1, see Additional file 1). Most contributions (23%) originate from *Bundesgesundheitsblatt*, followed by *Das Gesundheitswesen *(11%) and *Psychiatrische Praxis *(10%).

### Analysis of the journals

The instructions for authors were examined in all 31 journals using the freely accessible internet homepages of the journals (all accesses on July 10, 2006):

▪ Is a statement on conflicts of interest demanded?

▪ If so, do the instructions refer to the criteria of the International Committee of Medical Journal Editors (ICMJE criteria)?

▪ If they do not refer to the ICMJE criteria, which other criteria are applied?

### Analysis of the articles

Of the articles published in magazines which require an authors' statement on conflicts of interest (n = 71), we examined the following questions using full text articles:

▪ Is the statement explicitly published? We considered a statement as explicitly published if it was presented in the form of a specific paragraph with an explicit title "conflicts of interest" or likewise. We did not consider a statement as explicitly published if information on conflicts of interest could be gained from, e.g., the acknowledgement or unspecificly titled paragraphs (e.g. "annotations").

▪ If the statement is explicitly published, what kind of information is presented in the text?

▪ Are statements on conflicts of interest available from others who are essentially involved in the publication process (reviewers, editors)?

All articles (with and without explicit statements of conflicts of interest; differentiated in the tables 2 and 3, see Additional file 1) were examined as to whether information on possible conflicts of interest could be deducted from the institutional affiliations of the authors (e.g. staff members of for-profit companies) and from the acknowledgements.

Furthermore we wanted to gather information about the relevance of pharmaceutical topics within the included publications, since these topics are particularly important in respect to the research questions. For this purpose the titles and the summaries of all 124 articles were qualitatively analysed: does the article particularly deal with pharmaceutical topics?

The analyses of both the articles and the journals were performed by the principal investigator (NSCH) and the co-author (HL) independently from each other. A medical data assistant crossed-checked the findings. In case of different assessments the investigators came to a decision by consensus.

## Results

Of the included journals, 18 (58%) require an authors' statement on conflicts of interest, 13 (42%) do not require a statement (table 1, see Additional file 1). Of the 18 magazines which require a statement,

▪ two journals explicitly refer to the ICMJE criteria (*Psychiatrische Praxis *und *Wiener Klinische Wochenschrift*);

▪ 16 journals provide differently accentuated instructions on how to disclose conflicts of interest, whereby three of these journals exlusively focus on financial conflicts of interest, (*Zeitschrift für Orthopaedie und ihre Grenzgebiete, Deutsche Medizinische Wochenschrift, Fortschritt in der Neurologie-Psychiatrie)*. The remaining 13 journals include the issue of non-financial conflicts of interest in different ways, e.g. by phrases like "activities of a participant" or "organizational support".

71 articles are published in the 18 journals which require an authors' disclosure on conflict of interest (table 2, see Additional file 1). In 11 of these articles (15%) we found explicit publication of the statements; these articles are from following journals: *Deutsche Medizinische Wochenschrift *(n = 3), *HNO, Der Unfallchirurg, Der Schmerz, Der Anaesthesist, Der Orthopaede, Der Hautarzt, Der Internist, Der Nervenarzt *(in each case n = 1). Related to the total number of included articles, this means that the readers are informed of potential conflicts of interest in 9% of the cases (11 of 124 articles), independently of whether or not conflicts of interest exist. In 10 of the 11 statements no conflicts of interest are declared, (articles no. 4, 19, 26, 30, 33, 36, 40, 46, 64, 65). In a contribution to *Deutsche Medizinische Wochenschrift *it is indicated that the investigation was planned and evaluated by a drug company, and that the authors are staff members of this company (no. 22).

In five articles, topically relevant information can be gained from the institutional affiliation of the authors and the acknowledgements and annotations respectively:

▪ Himmel et al. point out that they received allowance from a pharmaceutical company for a part of their work; however, an influence on the study was ruled out by contract (article no. 29);

▪ the author of article no. 85 is director of a pharmaceutical company;

▪ two of ten authors of article no. 88 are staff members of a pharmaceutical company;

▪ Morgenroth et al. thank a pharmaceutical company for its support (no. 102);

▪ in article no 35 the authors declared no conflicts of interest in a paragraph titled "annotation"; however, this does not fulfill our critera for an explicit statement.

In none of the examined articles statements on conflicts of interests of others that are involved in the publication process (reviewers, editors) could be found.

As far as is recognizable from the titles and summaries, pharmaceutical topics are particularly dealt with in 26 of the 124 articles (21%). In less than a third of these articles information on conflicts of interest could be found, (8 of 26 articles: no. 22, 29, 33, 46, 56, 85, 88, 102), partly in the form of explicit disclosures and partly in the form of acknowledgements and the authors' institutional affiliations.

## Discussion

For the first time, this study examines the disclosure practice of conflicts of interest in German language publications on health services research. Some limitations need to be discussed. We confined our search to the PubMed data base. Therefore the results are limited by the fact that some journals which were not listed in PubMed at the date of search – but which could also be relevant in this context – were not included: for example, *Zeitschrift für Allgemeinmedizin *that has a high portion of articles on health services research and which publishers are known to be intensively engaged in the topic of conflicts of interests [13]. However, we assume that our findings would not significantly differ if we had used further data bases.

Furthermore, no English language articles were included, thus the data are not representative for all publications on health services research from Germany. However, it is assumed that the scientific debate on health services research topics in Germany has so far significantly taken place in German language journals.

### Little transparency

Overall, the sensitivity for the problem of conflicts of interest seems to be in need of improvement in the circle of the examined journals. Only about half of the journals expect statements from authors on conflicts of interest – and only in a few cases is the reader explicitly informed about the statements. Whether the received statements are published with the articles is in the responsibility of the editors, sometimes in agreement with the authors.

The transparency would be greater if the statements were published on principle, independently whether conflicts of interest are declared or not. This procedure would give the reader the chance to develop an unfiltered opinion about possible conflicts of interest; furthermore, this procedure could be suitable to increasing the sensitivity for the problem of all parties involved (authors, editors, reviewers, readers). Information on possible conflicts of interest of reviewers and editors are not available in any of the articles, although this could also be considered as good scientific practice [10]. Particularly little is known about editors' conflicts of interest and how to manage this problem [14].

### Insufficient consideration of non-financial conflicts of interest

Another critical issue is the understanding of conflicts of interest. The fact that they can result from direct financial allowances is comparatively self-evident. However, the various other causes for possible conflicts of interest are easily underestimated [11]. Thus the International Committee of Medical Journal Editors (ICMJE) describes the characteristics of non-financial interest conflicts in detail [10].

Of the German language journals included in this study, only two journals explicitly refer to the "uniform requirements for manuscripts submitted to biomedical journals". In the instructions for authors in the remaining 29 journals the topic of non-financial conflicts of interest is in no case dealt with as elaborately as the ICMJE criteria suggest. Instead, non-financial conflicts of interest are addressed in a very general way, and partially completely left blank.

The three journals with the most contributions on health services research in this study proceed differently (see table 1, see Additional file 1): *Bundesgesundheitsblatt *demands statements on conflicts of interest from the participants of the publication process (explicitly mentioned: authors, editors, reviewers), whereby financial aspects are addressed clearly and non-financial rather generally ("activities"). However, information on the statements is available only in one of the examined cases, in form of an acknowledgement (article no. 29). For the reader, this could mean that in all other cases no conflicts of interest were declared; it could also mean that conflicts of interest were declared but the responsible persons did not see the necessity to publish the statements. However, the readership has no chance to form an unfiltered opinion.

The journal *Das Gesundheitswesen *provides no information at all on the disclosure of conflicts of interest in its instructions for authors. *Psychiatrische Praxis *is one of the two journals which explicitly refer to the ICMJE criteria. However, in none of the 13 articles examined are the statements presented to the readership.

### Ambiguity of the understanding of conflicts of interest

The extent of ambiguity on the question of which issues should be declared as possible conflicts of interest may be illustrated by an example outside of the focus of this study. In the *Journal of the American Medical Association*, Kurt et al. published an article on "Migraine and risk of cardiovascular disease in women"; the authors declared that they have no relevant conflicts of interest [15].

In fact, the authors have numerous connections to pharmaceutical companies which sell products for the treatment of migraine and cardiovascular diseases, as Kurt et al. had to reveal later in a letter to the publishers. The authors state that they assumed that in the case of the article these connections are not conflicts of interest. They close with the sentence: "We have learned that it is best to disclose all relationships with for-profit companies and allow the editor(s) to decide what is relevant"[16]. In the editorial it is pointed out clearly that the editors of *JAMA *expect the disclosure of all possible conflicts of interest [17].

From the fact that only a fifth of the articles in our study deal with pharmaceutical topics in a closer or wider sense, it should not be concluded that conflicts of interest which may result from the influence of for-profit companies are less important in health services research than, e.g., in medical research. Besides the pharmaceutical industry, other for-profit companies could influence science, for example companies that sell technical equipment [18]. Furthermore, within the health system the influence on science of social insurance institutions, political institutions, various associations and numerous lobby groups has to be critically reflected – in particular against the background of the high proportion of commissioned studies that characterize health services research in Germany [12].

As in no other discipline health services research is concerned with the framework requirements of health care on different levels of the system. Thereby, various stakeholders could be very interested in using health services research to shape specific issues within the health care system in their favour. For example, one should reflect on the phenomenon of "disease mongering" which can be described as the attempt to define the population which allegedly suffers from a certain disease as large as possible and to extend the need for medical care [9]. A quotation from a critical editorial in *Zeitschrift für Allgemeinmedizin *makes clear how attentive health services research should be designed to avoid being used for the objectives of third parties: "develop a topic field, and prove that there is undersupply; everything else finds itself" [13].

### Implications for health services research in Germany

Pfaff and Kaiser recommend for future health services research in Germany that topics not in the immediate interest of financially potent stakeholders should specifically be promoted by the state [12]. Our data suggest that it is necessary to increase the transparency in the practice of scientific publication, not only related to direct financial allowances but also related to non-financial conflicts of interest. The "uniform requirements for manuscripts submitted to biomedical journals" [10] provide a good orientation for its realisation.

It is important that the claim for disclosure of all possible conflicts of interest contains no allegation against authors, editors or reviewers. An explicit comment seems to be useful at this point, e.g. as it is done in the instructions for authors of the journal *Der Nervenarzt *(table 1, see Additional file 1).

However, even if the disclosure practice is optimised, achieving full transparency in scientific publication is not a realistic ambition. In her editorial on the influence of money on science, DeAngelis outlined the problem as follows [1]: "There is simply no way to guarantee that all financial relationships and arrangements of all authors are disclosed. It is not feasible to independently investigate the financial relationships of every author, as no comprehensive, up-to-date source of this information exists". Finally the appeal for honesty remains the elementary component of good science.

## Conclusion

The study points out that a better sensitization for possible conflicts of interest in health services research is necessary. We suggest tightening the criteria for disclosure in the instructions for authors in the scientific journals. Among other things, this means

▪ similar consideration of financial and non-financial interest conflicts;

▪ obligatory publication of the statements to provide the readers with unfiltered information, independently of whether conflicts of interest are stated or not;

▪ application of this procedure to authors, reviewers and editors.

This strategy should be applied on all types of publications independent of their topics.

## Competing interests

The author(s) declare that they have no competing interests.

## Authors' contributions

NSCH conceived the study, conducted the systematic review, performed the analyses and drafted the manuscript. HL performed the analyses and helped to draft the manuscript. FWS helped to interpret the data and to draw the conclusions. All authors read and approved the manuscript.

## Appendix

^1 ^Our definition of health services research refers to the statement of the *Zentrum für Versorgungsforschung *(Centre of Health Services Research), Cologne, Germany [19].

## Pre-publication history

The pre-publication history for this paper can be accessed here:



## Supplementary Material

Additional data file 1 Click here for file
