# Supplementary material for: Disclosing conflicts of interest in German publications concerning health services research
Source: BMC Health Serv Res. 2007 Jun 1;7:78. doi: 10.1186/1472-6963-7-78 (PMC1905913; doi:10.1186/1472-6963-7-78)
Supplement: Additional data file 1 [file 1472-6963-7-78-S1.doc]

**Table 1:** German language journals with articles on health services research that were included in the study.

| **Journals` title** | **Number of articles included in the analysis** | **Do the journals demand a statement on conflicts of interest (according to the instructions for authors)?** | **If so, do the instructions for authors refer to the ICMJE criteria?** | **Uniform ressource locator of the journal (all accesses on July 10th, 2006)** |
| --- | --- | --- | --- | --- |
| Bundesgesundheitsblatt, Gesundheitsforschung, Gesundheitsschutz | 29 | yes | no | http://springerlink.metapress.com/content/1437-1588/ |
| Das Gesundheitswesen | 14 | no |  | <http://www.thieme.de/fz/gesu/index.html> |
| Psychiatrische Praxis | 13 | yes | yes | http://www.thieme.de/fz/psychiat-praxis/index.html |
| Medizinische Klinik | 9 | no |  | <http://www.urban-vogel.de/cda/DisplayContent.do?wid=100525> |
| Zeitschrift für Gerontologie und Geriatrie | 8 | no |  | <http://www.steinkopff.springer.de/portal/stk/index.htm> |
| Zeitschschrift für Arztliche Fortbildung und Qualitätssicherung | 6 | yes | no | http://www.urbanundfischer.de/ |
| Die Rehabilitation | 5 | no |  | http://www.thieme.de/fz/rehabilitation/index.html |
| Pflege | 5 | no |  | <http://verlag.hanshuber.com/ezm/index.php?ezm=PFL> |
| Zeitschschrift für Orthopädie und ihre Grenzgebiete | 5 | yes | no | http://www.thieme.de/fz/zfo/ |
| Deutsche Medizinische Wochenschrift | 3 | yes | no | <http://www.thieme.de/dmw/index.html> |
| Pflege Zeitschrift | 3 | no |  | <http://www.pflegezeitschrift.de/> |
| Der Nervenarzt | 2 | yes | no | http://springerlink.metapress.com/content/1433-0407/ |
| Journal der Deutschen Dermatologischen Gesellschaft (JDDG) | 2 | yes | no | http://www.blackwell-synergy.com/loi/ddg?cookieSet=1 |
| Psychotherapie, Psychosomatik, medizinische Psychologie | 2 | no |  | http://www.thieme-connect.de/ejournals/toc/ppmp |
| MMW Fortschritte der Medizin | 2 | no |  | http://www.urban-vogel.de/cda/DisplayContent.do?wid=100526 |
| Der Internist | 1 | yes | no | http://springerlink.metapress.com/content/1432-1289/ |
| HNO | 1 | yes | no | http://www.springerlink.com/content/1433-0458/ |
| Fortschritte der Neurologie-Psychiatrie | 1 | yes | no | http://www.thieme-connect.de/ejournals/toc/fdn |
| Zeitschrift für Kardiologie | 1 | no |  | http://springerlink.metapress.com/content/1435-1285/ |
| Forschende Komplementärmedizin und klassische Naturheilkunde | 1 | yes | no | http://content.karger.com/ProdukteDB/produkte.asp?Aktion=JournalHome&ProduktNr=224242&ContentOnly=false |
| Der Anaesthesist | 1 | yes | no | http://www.springerlink.com/content/1432-055X/ |
| Mund-, Kiefer- und Gesichtschirurgie | 1 | no |  | http://springerlink.metapress.com/content/1434-3940/ |
| Wiener medizinische Wochenschrift | 1 | no |  | http://www.springerlink.com/content/1563-258X/ |
| Wiener klinische Wochenschrift [The Middle European Journal of Medicine] | 1 | yes | yes | http://www.springer.com/dal/home/authors?SGWID=1-111-70-1087877-0 |
| Der Orthopäde | 1 | yes | no | http://springerlink.metapress.com/content/1433-0431/ |
| Zeitschrift für Gastroenterologie | 1 | yes | no | http://www.thieme.de/fz/zfg/index.html |
| Der Unfallchirurg | 1 | yes | no | http://www.springer.com/dal/home/generic/search/results?SGWID=1-40109-70-1002422-0 |
| Der Hautarzt | 1 | yes | no | http://www.springerlink.com/content/1432-1173/ |
| Der Schmerz | 1 | yes | no | http://springerlink.metapress.com/content/1432-2129/ |
| Anästhesiologie, Intensivmedizin, Notfallmedizin, Schmerztherapie: AINS | 1 | no |  | http://www.thieme-connect.de/ejournals/toc/ains |
| Praxis der Kinderpsychologie und Kinderpsychiatrie | 1 | no |  | http://www.v-r.de/de/zeitschriften/500024/ |

**Table 2:** Articles published in journals which demand a statement on conflicts of interest (according to the instructions for authors)

|  | **Authors** | Title of the article | **Journal** | **Statement on conflicts of interest explicitly published?*** |
| --- | --- | --- | --- | --- |
|  | Adler & Wolfersdorf | Zur Entwicklung der Inanspruchnahme von stationären psychotherapeutischen Leistungen an vollversorgenden Abteilungen und Fachkrankenhäusern für Psychiatrie und Psychotherapie in den neuen Bundesländern [*The Development of Utilisation of Psychuneurotic In-Patients in Hospitals for Psychiatry and Psychotherapy of the So-Called „New“ Federal States of Germany*] | Psychiatrische Praxis 2004; 31, Supplement 1; S79-S81 | no |
|  | Bäsler, Fuchs & Scriba | Förderung der Versorgungsforschung durch die Bundesärztekammer  [*The German Medical Association support initiative on health care research*] | Bundesgesundheitsblatt-Gesundheitsforschung-Gesundheitsschutz 2006: 49:130-136 | no |
|  | Bäthis et al. | Stellenwert von Endoprothetik und Umstellungsosteothomie bei Gonarthrose – Ergebnisse einer Umfrage an unfallchirurgischen und orthopädischen Kliniken [*Total Knee Arthroplasty and High Tibial Osteotomy in Osteoarthritis – Results of a Survey in German Hospitals*] | Zeitschrift für Orthopädie und Ihre Grenzgebiete 2005; 143: 19-24 | no |
|  | Beck et al. | Apparative Diagnostik im Schockraum [*Diagnostics and equipment in the shock trauma room*] | Der Unfallchirurg 2004: 107:862-870 | yes |
|  | Bender & Grouven | Möglichkeiten und Grenzen statistischer Regressionsmodelle zur Berechnung von Schwellenwerten für Mindestmengen [*Possibilities and limitations of statistical regression models for the calculation of threshold values for minimum provider volumes*] | Zeitschrift für Ärztliche Fortbildung und Qualitätssicherung 2006; 100: 93-98 | no |
|  | Bitzer et al. | Prozess- und ergebnisorientiertes Qualitätsmanagement in der Kurzzeitchirurgie mit Patientenbefragungen [*Patient reported process and outcome indicators for clinical audit in short-stay surgery*] | Zeitschrift für Ärztliche Fortbildung und Qualitätssicherung 2006; 100:189-195 | no |
|  | Bormann & Müller | Longitudinaldaten im Gesundheitsbereich [*Longitudinal data related to health. A survey of health related panel studies in Germany*] | Bundesgesundheitsblatt-Gesundheitsforschung-Gesundheitsschutz 2005; 48: 273-278 | no |
|  | Bormann | Gesundheitssurveys in Europa [*Health surveys in Europe. Overview and activities to harmonize the methods and instruments for comparing the data*] | Bundesgesundheitsblatt-Gesundheitsforschung-Gesundheitsschutz 2005; 48: 1383-1389 | no |
|  | Bramesfeld & Holler | Der Einfluss der Ausführungspraxis des Bundessozialhilfegesetzes auf die Deinstitutionalisierung psychisch Kranker [*The Impact of Social Welfare Policies on the Deinstitutionalisation of the Mentally Ill in Germany*] | Psychiatrische Praxis 2004; 31:387-394 | no |
|  | Brennecke et al | Therapie des Erysipels in Deutschland und Österreich – Ergebnisse einer Umfrage an deutschen und österreichischen Hautkliniken [*Treatment of erysipelas in Germany and Austria – Results of a survey in German and Austrian dermatological clinics*] | Journal der Deutschen Dermatologischen Gesellschaft 2005; 3:263-270 | no |
|  | Brodhun, Kramer & Krause | Befragung der Gesundheitsämter zur Umsetzung des Meldewesens nach dem Infektionsschutzgesetz [*Survey among local health departments concerning the implementation of the new infectious system*] | Bundesgesundheitsblatt-Gesundheitsforschung-Gesundheitsschutz 2004; 47: 755-761 | no |
|  | Brzank et al. | Häusliche Gewalt gegen Frauen und Versorgungsbedarf [*Domestic violence against women and health care demands. Results of a female emergency department patient survey*] | Bundesgesundheitsblatt-Gesundheitsforschung-Gesundheitsschutz 2005; 48: 337-345 | no |
|  | Burger & Tiemann | Diabetes mellitus in Deutschland. Eine Bestandsaufnahme nach Daten des telefonischen Gesundheitssurveys 2003 [*Diabetes mellitus in Gemany. Review of the situation according to the Telephone Health Survey 2003*] | Bundesgesundheitsblatt-Gesundheitsforschung-Gesundheitsschutz 2005; 48:1242-1249 | no |
|  | Dierks | Integrierte Versorgung aus juristischer Sicht [*Integrated care from a legal point of view*] | Zeitschrift für ärztliche Fortbildung und Qualitätssicherung 2006; 100: 37-39 | no |
|  | Diepgen | Demographische Entwicklung der Bevölkerung [*Demographic development of the population*] | Journal der Deutschen Dermatologischen Gesellschaft 2005; Supplement 2:S36-S39 | no |
|  | Dittrich et al. | Art und Umfang der Diagnostik bei der Akutbehandlung von Schlaganfallpatienten in neurologischen, internistischen und geriatrischen Abteilungen [*Differences in the Extent of Diagnostic Procedures after Acute Stroke in Patients Treated in Departments of Neurology, Internal Medicine and Geriatric Medicine*] | Fortschritt der Neurologie-Psychiatrie 2005; 73:68-73 | no |
|  | Ekkernkamp et al. | Anamnestische und klinische Befunde bei schweren Rückenschmerzen: eine klinisch epidemiologische Untersuchung an einer Stichprobe von LVA-Versicherten [*Physical Impairments and Comorbidities in a Sample of Members of a Pension Fund of Blue Collar-Workers Suffering from Severe Low Back Pain*] | Zeitschrift für Orthopädie und Ihre Grenzgebiete 2004; 142: 720-726 | no |
|  | Engel, Pötschke & Simonson | Telefonsurveys. Vor- und Nachteile [*Telephone surveys. Advantages and drawbacks*] | Bundesgesundheitsblatt-Gesundheitsforschung-Gesundheitsschutz 2005; 48:1217-1223 | no |
|  | Fellinger et al. | Psychosoziale Merkmale bei Gehörlosen. Daten aus einer Spezialambulanz für Gehörlose [*Psychosocial characteristics of deaf people: evaluation of data from a special outpatient clinic for the deaf*] | Der Nervenarzt 2005; 76:43-51 | yes |
|  | Geraedts | Versorgungsforschung in der operativen Medizin am Beispiel der Mammakarzinomchirurgie [*Health services research in surgery: the example of breast cancer surgery*] | Bundesgesundheitsblatt-Gesundheitsforschung-Gesundheitsschutz 2006; 449:160-166 | no |
|  | Grabe et al. | Seelische Belastung und Inanspruchnahme psychiatrischer und psychotherapeutischer Versorgung. Ergebnisse der Study of Health in Pomerania [*Mental Distress and the Use of Psychiatric and Psychotherapeutic Treatments Services. Results of the Study of Health in Pomerania (SHIP)* ] | Psychiatrische Praxis 2005; 32:299-303 | no |
|  | Glaab et al. | Leitlinienkonforme ambulante COPD-Behandlung in Deutschland [*Outpatient management of COPD in Germany according to national or international guidelines*] | Deutsche Medizinische Wochenschrift 2006; 131:1203-1208 | yes |
|  | Haberfellner et al. | Der Versorgungsbedarf ehemaliger psychiatrischer Langzeitpatienten [*Needs for Care of Former Long-Stay-Patients Living Outside of the Psychiatric Hospital*] | Psychiatrische Praxis 2006; 33:23-29 | no |
|  | Härtel und Volger | Inanspruchnahme und Akzeptanz klassischer Naturheilverfahren und alternativer Heilmethoden in Deutschland – Ergebnisse einer repräsentativen Bevölkerungsstudie [*Use and Acceptance of Classical Natural and Alternative Medicine in Germany - Findings of a Representative Population-Based Survey*] | Forschende Komplementärmedizin und Klassische Naturheilkunde | no |
|  | Heininger | Risiken von Infektionskrankheiten und der Nutzen von Impfungen [*Complications of infectious diseases and benefits of immunization*] | Bundesgesundheitsblatt-Gesundheitsforschung-Gesundheitsschutz 2004; 47:1129-1135 | no |
|  | Heinz & von Mallek | Vorkommnisse bei Hüft- und Knieendoprothesen [*Survey of incidents associated with hip and knee replacement devices. Analysis of the surveillance and registration system for medical products for the years 2000-2002*] | Der Orthopäde 2005; 34:47-54 | yes |
|  | Heudorf et al. | Hygiene beim Endoskopieren in Klinik und Praxis, 2003: Ergebnisse der infektionshygienischen Überwachung der Endoskopieeinrichtungen in Frankfurt am Main durch das Gesundheitsamt [*Hygiene in Endoscopy – Data on the Quality of Reprocessing Flexible Endoscopes and Endoscopic Accessories in Hospitals and Private Practices, 2003*] | Zeitschrift für Gastroenterologie 2004; 42:669-676 | no |
|  | Hewer, Salize & Wolfersdorf | Umfrage zur Qualität der internistischen Versorgung in psychiatrischen Fachkrankenhäusern [*Quality of Care in International Medicine in German Psychiatric Hospitals*] | Psychiatrische Praxis 2004; 31:404-408 | no |
|  | Himmel, Hummers-Pradier & Kochen | Medizinische Versorgung in der hausärztlichen Praxis [*Health services research in general practice. A new approach*] | Bundesgesundheitsblatt-Gesundheitsforschung-Gesundheitsschutz 2006; 49:151-159 | no |
|  | Himmelsseher & Werner | Therapeutische Hypothermie nach Schädel-Hirn-Trauma oder Subarachnoidalblutung [*Therapeutic hypothermia after traumatic brain injury or subarachnoid hemorrhage Current practices of German anaesthesia departments in intensive care*] | Der Anaesthesist 2004; 53:1168-1176 | yes |
|  | Holzinger, Matschinger & Angermeyer | Public-Mental-Health-Forschung im deutschen Sprachraum. Eine Analyse wissenschaftlicher Zeitschriften [*Research on Public Mental Health in the German-Speaking Area. An Analysis of Scientific Journals*] | Psychiatrische Praxis 2004; 31:369-377 | no |
|  | Horch & Wirtz | Nutzung von Gesundheitsinformationen [*People`s interest in health information*] | Bundesgesundheitsblatt-Gesundheitsforschung-Gesundheitsschutz 2005; 48:1250-1255 | no |
|  | Icks et al. | Versorgungsqualität und Ausmaß von Komplikationen an einer bevölkerungsbezogenen Stichprobe von Typ 2-Diabetespatienten. Der KORA-Survey 2000 [*Quality of care and prevalence of complications in a random population-based sample of patients with type 2 diabetes mellitus, based on the KORA Survey 2000*] | Deutsche Medizinische Wochenschrift 2006; 131:73-78 | yes |
|  | Ibach et al. | Die Situation der Angehörigenberatung bei Patienten mit frontotemporaler Demenz in der Gerontopsychiatrie [*The Situation of Caregiver Counselling in Patients with Frontotemporal Lobar Dementia in Old Psychiatry*] | Psychiatrische Praxis 2004; 31, Supplement 1:;S120-S122 | no |
|  | Isensee & Hanewinkel | Evaluation der Tabaksteuererhöhung von 1. Januar 2003 [*Evaluation of the 2003 tobacco tax increase in Germany*] | Bundesgesundheitsblatt-Gesundheitsforschung-Gesundheitsschutz 2004; 47:771-779 | no |
|  | Jecker, Melzer & Hitzler | Notwendigkeit der Bluttransfusion bei ausgewählten Operationen im Kopf-Hals-Bereich [*Necessiiy for blood transfusion in head and neck surgery*] | HNO 2005; 53:945-951 | yes |
|  | Klecha, Barke & Gureje | Die Versorgung psychisch Kranker in den Ländern der dritten Welt am Beispiel von Nigeria [*Mental health care in developing countries: the example of Nigeria*] | Der Nervenarzt 2004; 75:1118-1122 | no |
|  | Von dem Knesebeck et al. | Forschung zur Versorgung im höheren Lebensalter. Prävention, Case Management und Versorgung von Demenz [*Research on health care for the elderly. Prevention, case management, and care for patients with dementia*] | Bundesgesundheitsblatt-Gesundheitsforschung-Gesundheitsschutz 2006; 49:167-174 | no |
|  | Kohler, Rieck & Borch | Methode und Design des telefonischen Gesundheitssurveys 2003 [*Method and design of the Telephone Health Survey2003*] | Bundesgesundheitsblatt-Gesundheitsforschung-Gesundheitsschutz 2005; 48:1224-1230 | no |
|  | Komarahadi et al. | Verteilung von Schmerzparametern bei chronischen Schmerzpatienten im Vergleich zur Allgemeinbevölkerung [*Distribution of pain parameters for chronic pain patients in comparison to the general population*] | Der Schmerz 2006; 20:108-118 | yes |
|  | Krafft | Die EudraCT-Datenbank bei der EMEA zur Erfassung klinischen Prüfungen in Europa [*The community clinical trial system EudraCT at the EMEA fort he monitoring of clinical trials in Europe*] | Bundesgesundheitsblatt-Gesundheitsforschung-Gesundheitsschutz 2005; 48:453-458 | no |
|  | Kreutzer et al. | Zementierte Hüftendoprothetik in Deutschland – eine Update [*Cemented Total Hip Arthroplasty in Germany – An Update*] | Zeitschrift für Orthopädie und Ihre Grenzgebiete 2005; 143: 48-55 | no |
|  | Lampert & Burger | Verbreitung und Strukturen des Tabakkonsums in Deutschland [*Distribution and patterns of tobacco consumption in Germany*] | Bundesgesundheitsblatt-Gesundheitsforschung-Gesundheitsschutz 2005; 48:1231-1241 | no |
|  | Lange & Lampert | Die Gesundheit arbeitsloser Frauen und Männer [*The health of unemployed women and men. First results of the Telephone Health Survey 2003*] | Bundesgesundheitsblatt-Gesundheitsforschung-Gesundheitsschutz 2005; 48:1256-1264 | no |
|  | Lüring et al. | Gegenwärtige Praxis der Behandlung des Knorpelschadens am Kniegelenk – Ergebnisse einer deutschlandweiten Umfrage an unfallchirurgischen und orthopädischen Kliniken [*Current Treatment Modalities for Cartilage Defects at the Knee – Results of a Nation-Wide Survey of Trauma and Orthopaedic Clinics in Germany*] | Zeitschrift für Orthopädie und Ihre Grenzgebiete 2005; 143: 48-55 | no |
|  | Maywald et al. | Arzneimittelberatung für Patienten – Bedarfsanalyse, Evaluation und Einfluß auf die Compliance [*Drug information for patients – unmet needs, evaluation and influence on the compliance*] | Deutsche Medizinische Wochenschrift 2005; 130:1485-1490 | yes |
|  | Morfeld & Wirtz | Methodische Ansätze in der Versorgungsforschung. Das Beispiel Evaluation der Disease-Management-Programme [*Methods in health services research. The example of the evaluation of the German disease management programmes*] | Bundesgesundheitsblatt-Gesundheitsforschung-Gesundheitsschutz 2006; 49:120-129 | no |
|  | Offergeld er al. | Infektionsepidemiologische Daten von Blutspendern 2003-2004 [*HIV, HCV, HBV and syphilis infections among blood donors in Germany 2003-2004. Report of the Robert Koch Institute in accordance with Article 22 of the Transfusion Act*] | Bundesgesundheitsblatt-Gesundheitsforschung-Gesundheitsschutz 2005; 48:1273-1288 | no |
|  | Pfaff & Kaiser | Aufgabenverständnis und Entwicklungsstand der Versorgungsforschung. Ein Vergleich zwischen den USA, Großbritannien, Australien und Deutschland [*Tasks and development of health services research.. A comparison between the USA, UK, Australia and Germany*] | Bundesgesundheitsblatt-Gesundheitsforschung-Gesundheitsschutz 2005; 48:1273-1288 | no |
|  | Pudel & Ellrott | 50 Jahre Ernährungsaufklärung. Anmerkungen und Zukunftsperspektiven [*Fifty years of nutrition counseling. Remarks and perspectives*] | Bundesgesundheitsblatt-Gesundheitsforschung-Gesundheitsschutz 2004: 47:780-794 | no |
|  | Raspe | Versorgungsforschung. Das gemeinsame Programm von BMBF und GKV [*Health services research. A joint funding programme of Germany`s statutory health insurance funds and the Federal Ministry of Education and Research*] | Bundesgesundheitsblatt-Gesundheitsforschung-Gesundheitsschutz 2006; 49:137-140 | no |
|  | Reiter | Ausgewählte Daten zum Impf- und Immunstatus in Deutschland [*Vaccination coverage in Germany*] | Bundesgesundheitsblatt-Gesundheitsforschung-Gesundheitsschutz 2004; 47:1144-1150 | no |
|  | Roick et al. | Das Regionale Psychiatriebudget: Ein neuer Ansatz zur effizienten Verknüpfung stationärer und ambulanter Versorgungsleistungen [*The Regional Budget for Mental Health Care: A New Approach to Combine Inpatient and Outpatient Care*] | Psychiatrische Praxis 2005; 32:177-184 | no |
|  | Schulz et al. | Versorgungsforschung in der psychosozialen Medizin [*Health services research in psychosocial medicine*] | Bundesgesundheitsblatt-Gesundheitsforschung-Gesundheitsschutz 2006; 49:175-187 | no |
|  | Sagebiel er al. | Zukünftige Tuberkulinversorgung in Deutschland [*Future supply of tuberculin in Germany*] | Bundesgesundheitsblatt-Gesundheitsforschung-Gesundheitsschutz 2005; 48:477-482 | no |
|  | Saletu et al. | Diagnostik und Therapie des Restless-Legs-Syndroms in der Arztpraxis [*Diagnosis and therapy of restless legs syndrome in the doctor`s office*] | Wiener Klinische Wochenschrift 2004; 116/15-16:552-560 | no |
|  | Schneider et al. | Verdacht auf COPD – Entwicklung eines Algorithmus zur Stufendiagnostik in der Hausarztpraxis [*Suspicion of COPD – Development of an Algorithm for Stepwise Diagnosis in Primary Care*] | Zeitschrift für ärztliche Fortbildung und Qualitätssicherung 2005; 99:217-225 | no |
|  | Schneider, Dinant & Szecsenyi | Zur Notwendigkeit einer abgestuften Diagnostik in der Allgemeinmedizin als Konsequenz des Bayes`schen Theorems [*Stepwise diagnostic workup in general practice as a consequence of the Bayesian reasonig*] | Zeitschrift für ärztliche Fortbildung und Qualitätssicherung 2006; 100: 121-127 | no |
|  | Schrappe | Patientensicherheit im Krankenhaus als Gegenstand der Versorgungsforschung [*Patient safety in hospitals – a health services research issue*] | Bundesgesundheitsblatt-Gesundheitsforschung-Gesundheitsschutz 2006; 49:198-201 | no |
|  | Seidel et al. | Patienten- und Verbraucherberatungsstellen nach § 65b SGB V – Wege zu einer hochwertigen Informationsvermittlung und Beratung [*Patient and Consumer Information Centres According to § 65b Social Security Code V – Means for High-Quality Information Exchange and Counselling*] | Zeitschrift für ärztliche Fortbildung und Qualitätssicherung 2005; 99:397-403 | no |
|  | Spießl et al. | Evidenzbasiertes Klinikmanagement auf Grundlage der psychiatrischen Basisdokumentation [*Evidence-Based Hospital Management Considering Data from the Psychiatric Basic Documentation System*] | Psychiatrische Praxis 2004; 31, Supplement 1:S9-S11 | no |
|  | Spyra & Müller-Fahrnow | Rehabilitation als Gegenstand der Versorgungsforschung [*Rehabilitation as a subject of health services research*] | Bundesgesundheitsblatt-Gesundheitsforschung-Gesundheitsschutz 2006; 49:188-197 | no |
|  | Steinert & Kallert | Medikamentöse Zwangsbehandlung in der Psychiatrie [*Involuntary Medication in Psychiatry*] | Psychiatrische Praxis 2006; 33:e1-e12 | no |
|  | Strassburg et al. | Lebertransplantation zwischen Indikation und Spenderallokation [*Liver transplantation: Challenge of medical necessity and allocation*] | Der Internist 2004; 45:1233-1245 | yes |
|  | Strittmatter | Psychosoziale Betreuung von Patienten mit Hauttumoren in Zeiten der Diagnosis Related Groups (DRG) [*Psychosocial counseling of skin cancer patients in these times of diagnosis related groups (DRG)*] | Der Hautarzt 2004; 55:735-745 | yes |
|  | Tingart et al. | Die Therapie der Osteonekrose des Femurkopfes: Ergebnisse einer bundesweiten Umfrage [*Treatment of Osteonecrosis of the Femoral Head: Current Treatment Concepts in Germany*] | Zeitschrift für Orthopädie und Ihre Grenzgebiete 2004; 142:553-558 | no |
|  | Walter, Schneider & Bisson | Krankheitslast und Gesundheit im Alter. Herausforderungen für die Prävention und gesundheitliche Versorgung [*Morbidity and health in old age. A challenge for prevention and health care*] | Bundesgesundheitsblatt-Gesundheitsforschung-Gesundheitsschutz 2006; 49:537-546 | no |
|  | Watzke et al. | Rehabilitative Elemente bei der Behandlung von Menschen mit psychischen Störungen: werden sie bei der Entwicklung von Leitlinien ausreichend berücksichtigt? [*Rehabilitative Elements fort the Treatment of Patients with Mental Disorders: Are they Considered in the Development of Guidelines?*] | Psychiatrische Praxis 2005; 32:79-86 | no |
|  | Welschehold & Kraus | Einbeziehung von Bezirkskrankenhäusern in die Entwicklung Gemeindepsychiatrischer Verbünde [*Integration of District Psychiatric Hospitals into the Development of Regional Community Psychiatry Networks – The Actual State Results of a Survey Among Medical Directors of Bavarian District Hospitals*] | Psychiatrische Praxis 2004; 31, Supplement 1:S12-S14 | no |
|  | Wiegand et al. | Wer braucht was? Neue Ansätze der multidisziplinären Diagnostik und Therapie adipöser Kinder und Jugendlicher in einer multiethnischen Großstadt [*Who needs what? New approaches to multidisciplinary diagnostics and therapy for adipose children and youths in a multiethnic city*] | Bundesgesundheitsblatt-Gesundheitsforschung-Gesundheitsschutz 2005; 48:307-314 | no |
|  | Wolfersdorf, Klein & Dose | Psychotherapiestationen in den bayerischen Bezirkskrankenhäusern [*Psychotherapy Units in Bavarian State Mental Hospitals*] | Psychiatrische Praxis 2004; 31, Supplement 1:S76-S78 | no |

* Criteria: statement presented in form of a specific paragraph with an explicit title “conflicts of interest” or likewise. We did not consider a statement as explicitly published if information on conflicts of interest could be gained from, e.g., the acknowledgement or unspecificly titled paragraphs (e.g. annotations).

**Table 3:** Articles published in journals which demand no statement on conflicts of interest (according to the instructions for authors)

| . | **Authors** | **Titel** | **Journal** |
| --- | --- | --- | --- |
|  | Allgeier & Kämmerle-Hofrichter | Studie zur Ermittlung des Unterstützungsbedarfs von Patientinnen und Patienten, die nach einem Schlaganfall zu Hause leben [*Determinig the support needs of patients who live at home following a stroke*] | Pflege 2005; 18:373-380 |
|  | Albrecht & Bramesfeld | Das Angebot an gemeindenahen beruflichen Rehabilitationsmöglichkeiten für psychisch kranke Menschen in der Bundesrepublik [*Facilities Offered by Community-Oriented Professional Rehabilitation Centres for Mentally Challenged Persons in the Federal Republic of Germany*] | Das Gesundheitswesen 2004; 66:492-498 |
|  | Bermejo, Bursch & Muthny | Subjektive Theorien zum Herzinfarkt im transkulturellen Vergleich [*Lay Theories Regarding Myocardial Infarction in a Transcultural Comparison*] | Psychotherapie, Psychosomatik, Medizinische Psychologie 2006; 56:3318-324 |
|  | Bäuerle, Specht-Leible & Voß | Veränderungen des Hilfe- und Pflegebedarfs nach hüftnahen Frakturen im höheren Lebensalter [*Hip fracture – changes in need of help and care – cluster analysis – formal and informal support*] | Zeitschrift für Gerontologie und Geriatrie 2004; 37:351-353 |
|  | Bestehorn | Medizinische Register: ein Beitrag zur Versorgungsforschung [*Medical Registries*] | Medizinische Klinik 2005; 100:722-728 |
|  | Brennecke | Anforderungen an eine zukunftsorientierte Sozialmedizin [*Requirements of a Future-oriented Social Medicine*] | Das Gesundheitswesen 2005; 67:81-88 |
|  | Christen et al. | Erfahrungen und Resultate von standardisierten Beobachtungen konventioneller und kinästhestischer Pflege auf einer radioonkologischen Abteilung [*Experiences and results from standardised observations of conventional and kinaesthetic nursing in a nuclear and radio-therapeutic ward*] | Pflege 2005; 18:25-37 |
|  | Dietzel-Papakyriakou | Potenziale älterer Migranten und Migrantinnen [*Potentials of elderly migrants in Germany*] | Zeitschrift für Gerontologie und Geriatrie 2005; 38-396-406 |
|  | Eberl, Bartholomeyczik & Donath | Die Erfassung des Pflegeaufwandes bei Patienten mit der medizinischen Diagnose Myokardinfarkt [*The amount of nursing care for patients with myocardial infarction – A descriptive study*] | Pflege 2005; 18:364-372 |
|  | Ehlebracht-König & Bönisch | Stationäre Nachsorge bei rheumatischen Erkrankungen – Konzept, Erprobung und Akzeptanz [*Inpatient Booster Group Treatment in Rheumatic Diseases: Concept, Trial, and Acceptance*] | Rehabilitation 2004; 43:358-367 |
|  | Erler & Fuchs | Praxisbezug in der sozialmedizinischen Lehre unter den Bedingungen der neuen ärztlichen Approbationsordnung [*An Example for a Practice-Oriented Curriculum in Social Medicine under the Conditions of the New Medical Licensing Regulations – Experiences with Practice-Oriented Teaching and Possibilities for Including Practical Issues into the Teaching Syllabus of Social Medicine after the Introduction of the New Medical Licensing Regulations*] | Das Gesundheitswesen 2005; 67:355-360 |
|  | Fuchs | Defizite und Perspektiven einer geschlechtergerechten Gesundheitsversorgung an den Beispielen KHK und psychische Störungen [*Deficits and Outlook on Gender-Specific Health Care as Exemplified by CHD and Mental Disorders*] | Das Gesundheitswesen 2005; 67: 124-128 |
|  | Fürstenberg, Heumann & Roeder | Auswirkungen von Mindestmengen auf die stationären Versorgungsstrukturen der Kardiologie [*Effects of minimum volume regulations on the provision of health care services in cardiology*] | Zeitschrift für Kardiologie 2005; 94:95-109 |
|  | Götte | Kostendämpfung und ihre Auswirkungen auf die Arzneimittelforschung [*Cost Reductions and their Effects on Drug Research*] | Medizinische Klinik 2005; 100:309-313 |
|  | Hanewinkel et al. | Motivierende Gesprächsführung mit Arbeitslosen [*Motivational Interviewing of Unemployed – Acceptance and Effects of Counselling to improve Health-Related Behaviour*] | Das Gesundheitswesen 2006; 68:240-248 |
|  | Hach et al. | Arzneimittelberatungsdienste als Instrumente der Versorgungsforschung [*Drug Information Centers – Instruments for Health Care Research?*] | Medizinische Klinik 2005; 100:396-400 |
|  | Heinen-Kammerer et al. | Versorgungssituation von Demenzkranken kann durch Ginkgo biloba verbessert werden. Ergebnisse einer Studie zur Versorgungsforschung hinsichtlich der Leistungsfähigkeit der Demenzkranken, der Lebensqualität der Pflegenden und der Gesamtkosten der Behandlung [*The Situation of Patients with Dementia may be Rectified by Ginkgo Biloba. Results of a Health Services Research Study Concerning the Ability of Patients with Dementia, Quality of Life of the Nursing Familiy Members and Total Treatment Costs*] | MMW Fortschritte der Medizin 2005; 147:127-133 |
|  | Hieber et al. | Die Übereinstimmung von Wohnbedürfnissen und Wohnbedingungen und ihr Einfluss auf die erlebte Stadtteilverbundenheit [*The fit of housing needs and housing conditions and its impact on outdoor place attachment* ] | Zeitschrift für Gerontologie und Geriatrie 2005; 38:293-300 |
|  | Hoffmann et al. | Zur Behandlungssituation erwachsener Patienten mit angeborenen Stoffwechselkrankheiten [*Situation of Adult Patients with Inborn Errors of Metabolism. A Survey in Germany*] | Medizinische Klinik 2005; 100:617-623 |
|  | Jäckel & Farin | Qualitätssicherung in der Rehabilitation: Wo stehen wir heute? [*Quality Assurance in Rehabilitation: Where Do We Stand Today?*] | Rehabilitation 2004; 43:271-283 |
|  | Janus & Amelung | Integrierte Versorgungssysteme in Kalifornien – Erfolgs- und Misserfolgsfaktoren der ersten 10 Jahre und Impulse für Deutschland [*Integrated Delivery Systems in California – Ten Years of Experience and Implications for Germany*] | Das Gesundheitswesen 2004; 66:649-655 |
|  | Jenull-Schiefer | ,,Geri-Aktiv“ – Die Aktivierung von Pflegeheimbewohnern [*„Geri-Aktiv“ – the activation of nursing home residents*] | Zeitschrift für Gerontologie und Geriatrie 2004; 37:360-362 |
|  | Josat | Welche Qualitätskriterien sind Angehörigen in der stationären Pflege wichtig? [*Which quality criteria are important for relatives in a nursing home? A single case study*] | Pflege 2005; 18:169-175 |
|  | Klünder | Alt und pflegebedürftig – den Lebensabend selbstbestimmt gestalten: Voraussetzungen und Grenzen in der häuslichen Versorgung [*Aged and in need of care – to shape one`s retirement self-determinedly: Pre-conditions and bounds of home care*] | Pflegezeitschrift 2005; 8:2-8 |
|  | Klie | Würdekonzept für Menschen mit Behinderung und Pflegebedarf, Balancen zwischen Autonomie und Sorgekultur [*Dignity for the frail old*] | Zeitschrift für Gerontologie und Geriatrie 2005; 38:268-272 |
|  | Köhlen & Friedemann | Überprüfung eines Familien-Assessment-Instruments auf der Grundlage der Theorie des systemischen Gleichgewichts [*Verification of a family assessment instrument based on the framework of systemic organisation*] | Pflege 2006; 19:23-32 |
|  | Krauth et al. | Empirische Bewertungsansätze in der gesundheitsökonomischen Evaluation – ein Vorschlag der AG Methoden der gesundheitsökonomischen Evaluation (AG MEG) [*Empirical Standard Costs for Health Economic Evaluation in Germany – a Proposal by the Working Group Methods in Health Economic Evaluation*] | Das Gesundheitswesen 2005; 67:736-746 |
|  | Krauth et al. | Gesundheitsökonomische Evaluation von Rehabilitationsprogrammen im Förderschwerpunkt Rehabilitationswissenschaften [*Health Economic Evaluation of Rehabilitation Programmes in the „Rehabilitation Sciences“ Research Funding Programme in Germany*] | Das Gesundheitswesen 2005; 44:e46-e56 |
|  | Leistner & Bublitz | Geriatrische Rehabilitation in der Bundesrepublik Deutschland: Versorgungspolitische und strukturelle Aspekte aus Sicht der gesetzlichen Krankenversicherung (GKV) [*Geriatric Rehabilitation in Germany: Service Provision Policy and Structural Aspects from a Statutory Health Insurance Perspective*] | Rehabilitation 2004; 43:296-303 |
|  | Lenz | Vorstellung der Kinder über die psychische Erkrankung ihrer Eltern. Eine explorative Studie [*Children's ideas about their parents' psychiatric illness--an explorative study* ][] | Praxis der Kinderpsychologie und Kinderpsychiatrie 2005; 54:382-398 |
|  | Morgenroth et al. | Die Durchimpfung von 24-30 Monate alten Kindern in pädiatrischen Praxen im Zeitraum von November 1999 bis Mai 2001 – Der Einfluß soziodemografischer Faktoren [*The Vaccination Coverage Among Children Aged 24-30 Months in Pediatric Offices November 1999-May 2001. The influence of Socio-demographic Characteristics*] | Das Gesundheitswesen 2005; 67:788-794 |
|  | Martini et al. | Eigenblutspende in der Dysgnathiechirurgie [*Preoperative autologous blood donation in orthognathic surgery*] | Mund Kiefer Gesichtschirurgie 2004; 8:376-380 |
|  | Meyer | Das personengebundene Budget bei Pflegebedürftigkeit. Systemwechsel eröffnet Chancen für Anbieter und Nachfrager [*Personal budget for persons in need of care A socio-economic change with new perspectives for supply and demand*] | Pflegezeitschrift 2005; 11: 2-8 |
|  | Mittendorf et al. | Arztkontaktpreise bei der ambulanten Versorgung der chronischen Polyarthritis in Deutschland [*Costs of Ambulatory Care for RA Patients in Germany*] | Medizinische Klinik 2005; 100:255-261 |
|  | Mohrmann et al. | Qualitätsprüfungen ambulanter Pflegedienste in Baden-Württemberg – Überblick über die Erfahrungen aus 6 Jahren flächendeckender Untersuchungen durch den MDK [*Quality Assurance of Outpatients Nursing Services in Baden-Württemberg – Overall View of 6 Years of Total Survey by the Medical Services of the Statutory Health Insurance (MDK)*] | Das Gesundheitswesen 2005; 67:694-700 |
|  | Prüß et al. | Verläufe von Pflegebedürftigkeit in Hessen in den Jahren 1999 bis 2002. Ergebnisse einer Längsschnittstudie [*Path ways through care in Hessen from 1999 to 2002. Results of a longitudinal study*] | Das Gesundheitswesen 2006; 68:123-127 |
|  | Pietsch & Schaffranietz | Anästhesiologisches Vorgehen bei orthotopen Lebertransplantationen (LTX) – Ergebnisse einer Umfrage [*Anaesthesiological Management in Orthotopic Liver Transplantation – Results of a Survey*] | Anästhesiologie, Intensivmedizin, Notfallmedizin, Schmerztherapie 2006; 41:21-26 |
|  | Raspe et al. | Bedarf an rehabilitativen Leistungen: Theorie und Empirie [*Being in Need of Rehabilitation Services: Concept and Data* ] | Rehabilitation 2005; 44:325-334 |
|  | Rau, Mensing & Brand | Medizinische Notfalldienste aus Nutzersicht. Ein Beitrag zur Versorgungsforschung durch CATI-Befragung der Bevölkerung [*„Medical Emergency Services as Seen by Consumers“. Health Services Research by CATI Survey of the Community*] | Medizinische Klinik 2006; 101:37-47 |
|  | Reiter & Haas | Das Risiko einer Pandemie wird immer größer [*Influenza Pandemic: A Real Threat?*] | MMW Fortschritte der Medizin 2005; 9: 35-38 |
|  | Schellevis, Westert & De Bakker | Die aktuelle Rolle der Allgemeinmedizin im niederländischen Gesundheitsversorgungssystem [*The Actual Role of General Practice of in the Dutch Health-Care System. Results of the Second Dutch National Survey of General Practice*] | Medizinische Klinik 2005; 100:656-661 |
|  | Schilder | Abbildung der Pflegebedürftigkeit mithilfe von Pflegediagnosen: Die NANDA-Klassifikation und ISNP differenziert betrachtet [*To represent needs of nursing care using nursing diagnoses: potentials and restrictions of the NANDA Classification and ICNP*] | Pflegezeitschrift 2005; 3:2-8 |
|  | Schneider et al. | Konzepte zur bedarfsgerechten Strukturierung der Palliativversorgung im deutschen Gesundheitswesen: das Beispiel des Bundeslandes Niedersachsen [*Concepts for a Demand-oriented Structuring of Palliative Care in the German Health Care System: The Example of Lower Saxony*] | Das Gesundheitswesen 2005; 67:755-762 |
|  | Schopf & Naegele | Alter und Migration – ein Überblick [*Age and ethnicity – an overview*] | Zeitschrift für Gerontologie und Geriatrie 2005; 38:384-395 |
|  | Schröder et al. | Inhousebefragung zur Patientenzufriedenheit in einem Klinikum der Maximalversorgung – Ein Praxisbericht [*In-House-Questioning on Patient Satisfaction in a General Maximum Care Hospital Complex – Report from Practice*] | Das Gesundheitswesen 2004; 66:674-681 |
|  | Schütte, Kirch & Walter | Versorgungsforschung in Deutschland – eine Standortbestimmung aus Sicht der Zahn-, Mund- und Kieferheilkunde [*Health Services Research in Germany – Status Quo in the Field of Oral Health* ] | Medizinische Klinik 2005; 100:562 |
|  | Schwarz & Wendel | Erwachsene mit angeborenen Stoffwechselkrankheiten. Eine neue Herausforderung für die Innere Medizin [*Inborn Errors of Metabolism (IEM) in Adults. A New Challenge to Internal Medicine (Part 2)*] | Medizinische Klinik 2005; 100:624-635 |
|  | Schubert | Flächendeckende Zahnprophylaxemaßnahmen und Feststellung erhöhter Bedarfe an Prophylaxemaßnahmen in Braunschweig 2003/2004 [*Area-wide Tooth-prophylaxis Methodes and Locating Increased needs in Prophylaxis in Braunschweig 2003/2004*] | Das Gesundheitswesen 2005: 67:145-149 |
|  | Sitta et al. | Faires Benchmarking der Behandlungsdauer depressiver Patienten in psychiatrisch-psychotherapeutischen Kliniken [*Duration of Inpatient Depression Treatment – Fair Benchmarking Between Hospitals*] | Psychotherapie, Psychosomatik, Medizinische Psychologie 2006; 56:128-137 |
|  | Thoma, Zank & Schacke | Gewalt gegen dementiell Erkrankte in der Familie: Datenerhebung in einem schwer zugänglichen Forschungsgebiet [*Domestic abuse of dementia patients: data collection in a difficult research area*] | Zeitschrift für Gerontologie und Geriatrie 2004; 37: 349-350 |
|  | Voigt-Radloff, Schochat & Heiß | Kontrollierte Studien zur Wirksamkeit von Ergotherapie bei Älteren [*Controlled trials on the efficacy of occupational therapy with elderly. Part I: Research question, search strategy and methodological quality of trials* ] | Zeitschrift für Gerontologie und Geriatrie 2004; 37:444-449 |
|  | Voß & Lohff | Die Kategorie Geschlecht und die daraus resultierenden Anforderungen an die medizinische Ausbildung [*The category gender and meeting standards for medical education as a result*] | Wiener Medizinische Wochenschrift 2004; 154/17-18: 394-403 |
|  | Zwingmann et al. | Förderschwerpunkt ,,Rehabilitationswissenschaften“: Ergebnisse – Umsetzung – Erfolge und Perspektiven [*The „Rehabilitation Sciences“ Research Funding Programme: Research Findings – Implementation – Impact and Perspectives*] | Rehabilitation 2004; 43:260-270 |
